# Optimization of the heavy metal (Bi–W–Gd–Sb) concentrations in the elastomeric shields for computer tomography (CT)

**DOI:** 10.1007/s10967-014-2985-5

**Published:** 2014-01-31

**Authors:** Piotr Szajerski, Marian Zaborski, Henryk Bem, Wlodzimierz Baryn, Edyta Kusiak

**Affiliations:** 1 Institute of Applied Radiation Chemistry, Lodz University of Technology, Wroblewskiego 15, 90-924 Lodz, Poland; 2 Institute of Polymer and Dye Technology, Lodz University of Technology, Stefanowskiego 12/16, 90-924 Lodz, Poland

**Keywords:** X-ray florescence radiation, Dose reduction, Shielding composites, Heavy metal additives, CT, Elastomer shields

## Abstract

Eight elastomeric composites (NRU, GR1–GR4, NRBG08–NRBG24) containing mixtures of different proportions of heavy metal additives (Bi, W, Gd and Sb) have been synthesized and examined as protective shields. The NRU sample was a pure rubber matrix and served as a reference sample for heavy metal modified composites. Experimental procedure used for evaluation of the composite shields and their attenuation properties was based on the utilization of HPGe spectrometry and analysis of X-ray fluorescence radiation intensity of the heavy metal additives in the following energy ranges for: Sb (20–35 keV), Gd (35–55 keV), W (55–70 keV) and Bi (70–90 keV). The main contributor to the induced X-ray fluorescence radiation within the shield is Bi additive and the intensity of the X-ray radiation generated within the energy range of 70–90 keV strongly depends on its concentration. It was found that decreasing concentration of the Bi fraction from 0.35 (GR samples) to 0.15 (NRBG samples) results in significant lowering Bi X-ray fluorescence radiation within the 70–90 keV energy range. Secondary effect of decreasing Bi concentration was efficient diminishing excitation processes for lower Z heavy metal additives (W, Gd and Sb, GR vs. NRBG samples). As the final quality parameter of the shielding properties for the examined elastomers, dose reduction factor (DRF) coefficients were calculated for each shield. It was found, that the best shielding properties are observed for composites with lower Bi concentration (0.15 vs. 0.35 Bi mass fraction) with only slight further improvement of their parameters (DRF) with increasing of Gd concentration (Gd mass fraction 0.08, 0.16 and 0.24). The most efficient dose reduction composite was found to be NRBG24 elastomer with DRF value 0.47 (53 % dose reduction) for ca. 2 mm and 0.44 g/cm^2^ layer thickness.

## Introduction

Computed tomography (CT) has become a key medical examination technique for patient management due to its outstanding diagnostic capabilities. However, the use of X-ray radiation is resulting in the slightly enhanced effective doses even in single CT examination, ranging from 2.5 to dozen mSv, in comparison to those from natural ionizing radiation sources (world average value of 2.4 mSv). The recent well statistically based epidemiological studies undoubtedly proved a positive association between the radiation dose from CT scans and leukaemia and other tumours in children [1, 2]. Therefore, the dose reduction in CT has become a top priority for all radiological practices [3].

Apart from a spectacular progress in the development of the new generation CT machines with reduced radiation exposure and improvement in the CT standardized protocols, which also substantially decreases the doses during these examinations [4], the use of the bismuth containing elastomeric shields for these purpose has been also recently strongly recommended [5]. A review of published dosimetry investigations of bismuth shield techniques has been reported by Kim et al. [6]. As it is evident from that review, several studies demonstrated that bismuth shields can effectively reduce the radiation dose without degrading image quality and it is a valuable tool to reduce radiation risk in children.

A bismuth shield primary function is to remove the lower energy photons contributing to the doses in the surface tissues. However, the use of the bismuth shields has been questioned because of the emission of the scattered additional photons from such shield, which may influence on the quality of the CT scans [7, 8]. One of the important source of such scattered radiation are fluorescence photons coming from excitation of heavy metal atoms after absorption of the incoming X-ray radiation [9]. The fluorescence radiation from the bismuth atoms can be reduced by an addition of the second metal additive with slightly lower atomic number, for example tungsten. However, tungsten after excitation emits fluorescence radiation with photon energies in the range of 60 keV, which may also influence both: on the quality of CT scans and surface tissue doses. Therefore, sometimes a third metal components, particularly gadolinium is added to the elastomeric shields.

The objective of our present study was to evaluate the optimal metal concentration of Bi, W and Gd in the rubber composites in order to achieve the highest dose reduction factor (DRF) for such shields.

## Materials and methods

The eight new types of elastomeric composites have been synthesized to evaluate the relation between heavy metal fraction and attenuation efficiency of the X-ray radiation by the shields. Especially the influence of the Gd concentration on shielding properties of the shields was investigated. For these purposes, eight different samples of elastomeric shields containing Bi (15–35 %), W (15 %), Gd (0–24 %) and Sb (3 %) have been prepared.

All samples of the elastomeric composites have been prepared after vulcanisation of the natural rubber and heavy metal oxides of Bi_2_O_3_, WO_3_, Gd_2_O_3_ and Sb_2_O_3_. The samples were synthesized in Institute of Polymer and Dye Technology of Lodz University of Technology. The chemicals used for synthesis were of analytical grade purity and used as obtained. The procedure was similar to that described previously elsewhere [9, 10]. The detailed composition of the synthesized elastomeric composites are presented in Table 1. Samples were prepared in the form of 140 × 80 mm^2^ sheets and ca 1 mm thickness. The shielding properties evaluation procedure was based on the measurement of the photons intensity out coming from the shield in the different energy spectrum ranges. These photons were induced within the shield by heavy metal atoms (Bi, W, Gd and Sb) excitation from the external Co-57 source. The measurements were taken within the photon energy range of 20–140 keV.

**Table 1:** Mass fractions of elastomeric composites, phr

Sample	NR (g)	ZnO (phr)	Sulphur (phr)	MBT (phr)	Stearic acid (phr)	Bi (Bi_2_O_3_) (phr)	W (WO_3_) (phr)	Gd (Gd_2_O_3_) (phr)	Sb (Sb_2_O_3_) (phr)
NRU	100	5	1	2	2	–	–	–	–
GR1	100	5	1	2	2	62.8 (70.0)	–	–	–
GR2	100	5	1	2	2	90.9 (101.3)	39.0 (49.2)	–	–
GR3	100	5	1	2	2	117.1 (130.5)	50.3 (63.4)	27.5 (31.7)	–
GR4	100	5	1	2	2	132.8 (148.1)	57.0 (71.9)	31.2 (36.0)	12.4 (14.8)
NRBG08	100	5	1	2	2	32.0 (35.67)	32.0 (40.3)	17.2 (19.8)	6.6 (7.8)
NRBG16	100	5	1	2	2	39.1 (43.6)	39.1 (49.3)	41.8 (48.1)	8.3 (9.9)
NRBG24	100	5	1	2	2	50.1 (55.8)	50.0 (63.0)	80.7 (93.0)	10.6 (12.7)

*NR* natural rubber, *MBT* 2-mercaptobenzothiazole, *phr* parts per hundred rubber

The radiation source used for experimental procedure was Co-57 closed isotopic source (POLATOM, Poland), emitting two main groups of photons of energy 122.1 (85.6 % emission probability) and 136.5 keV (10.7 %). Radiation detection system used was a coaxial HPGe detector (Canberra, GX3020) with thin beryllium window, housed in 10 cm thick lead shield lined inside with 1 mm of copper. The resolution of the detector was 0.9 keV for the 122.1 keV peak, and its relative efficiency was 30 % for the 1.33 MeV peak. The data were recorded (each spectrum over 1,800 s) and processed using Genie 2000 software from Canberra. Details of the detection system are described elsewhere [11]. The recorded spectra were quantitatively analyzed within the following energy ranges 20–35, 35–55, 55–70, 70–90 and 90–140 keV. The energy ranges were chosen according to the occurrence of the main X-ray fluorescence photon emission ranges of Sb, Gd, W and Bi respectively [12, 13], and Co-57 emission for the range 90–140 keV. Relative intensity of the X-ray fluorescence emission photons was generally weak comparing with the main 122.1 and 136.5 keV photons of Co-57, so for the weak X-ray fluorescence signals it was necessary to perform correction for the background radiation. Prior to the measurements of the protective shields, detection system and method were evaluated according to the procedure based on the measurements of the standardized lead plates described previously elsewhere [9]. The relative error of the method was ~1 %. Mass attenuation coefficients were calculated taking into account composition of investigated samples. Calculations were performed using XCom software available from the National Institute of Standards and Technology (NIST) website [14, 15]. The composition data for the human tissue (soft) used (H 10.20 %, C 14.30 %, N 3.40 %, O 70.80 %, Na 0.20 %, P 0.30 %, S 0.30 %, Cl 0.20 %, K 0.30 %) to calculate the mass attenuation coefficient and dose reduction factor were also taken from the NIST database [16] and based on the data included in the ICRU 44 report [17]. Next to the measurements of the X-ray fluorescence radiation, X-ray attenuation properties of the investigated composites were measured according to the PN-EN 61331-1:2003 standard by determination of their lead equivalents. The comparative kerma rate in air measurement method was applied using standardized lead foil as a reference material. As a X-ray source Gulmay X-ray Calibration System 300 kV was used. Samples were measured for 4 X-ray photons energies, 45, 57, 79 and 104 keV of ~50 % relative width at 1.5 m distance from the X-ray source. Air kerma rate was measured with ionization chamber (open type, model M23361, PTW Freiburg) connected to a reference electrometer UNIDOS E (T 10008 type, PTW Freiburg). Air kerma rate determination uncertainty was approximately about 2 %. Lead equivalents for the investigated composites were determined by comparison of attenuation factors obtained for the measured samples with reference curves generated using the standardized lead foil.

## Results and discussion

The main aim of the presented studies was optimization of the heavy metal concentrations in the protective shields previously described by us [9]. The main concept of the elastomeric shields for CT examination was utilization of the heavy metal additives with gradually decreasing atomic number, capable to attenuate efficiently X-ray fluorescence radiation generated within the shield itself. Metal concentration data of the elastomeric shields are presented in Table 1 (by components used during synthesis) and in Table 2 (by element, recalculation based on the data in Table 1). Two sets of composites were used in experimental procedure. The GR series (GR1–GR4), and NRBG series with constant Bi, W and Sb concentrations and gradually increasing fraction of Gd additive. Additionally, beside of the GR and NRBG composites, the raw sample of the pure rubber matrix were used (NRU) for comparison.

**Table 2:** Elemental composition of the Bi–W–Gd–Sb composite shields

Sample code	Thickness (cm)	Thickness (g/cm^2^)	Density (g/cm^3^)	Mass fraction									
				H	C	N	O	S	Zn	Sb	Gd	W	Bi
NRU	0.098	0.097	0.997	0.1093	0.8176	0.0015	0.0099	0.0252	0.0365	–	–	–	–
GR1	0.093	0.133	1.439	0.0668	0.4996	0.0009	0.0461	0.0154	0.0223	–	–	–	0.3488
GR2	0.105	0.0198	1.884	0.0462	0.3452	0.0006	0.0833	0.0106	0.0154	–	–	0.1498	0.3488
GR3	0.098	0.219	2.248	0.0358	0.2680	0.0005	0.0949	0.0082	0.0120	–	0.0820	0.1498	0.3488
GR4	0.093	0.218	2.357	0.0316	0.2362	0.0004	0.1009	0.0073	0.0105	0.0325	0.0820	0.1497	0.3489
NRBG08	0.115	0.191	1.658	0.0563	0.4210	0.0008	0.0797	0.0130	0.0188	0.0307	0.0804	0.1497	0.1498
NRBG16	0.105	0.203	1.932	0.0461	0.3447	0.0006	0.0912	0.0106	0.0154	0.0317	0.1601	0.1498	0.1499
NRBG24	0.094	0.220	2.343	0.0359	0.2688	0.0005	0.1025	0.0083	0.0120	0.0318	0.2412	0.1493	0.1496

Generation of the X-ray fluorescence radiation within the shields used for CT examination can be described by the first order linear differential equation (Eq. 1) [9, 12, 13, 18].

1 $$ {\text{d}}R_{\text{F}} \; = \;\omega Bc_{Me}\left( {\sum\limits_{i} {R_{\gamma i}^{{^{\text{o}} }} } e^{{ - \mu_{i} x}} \tau_{i} } \right){\text{d}}x - R_{\text{f}} \mu_{\text{f}} {\text{d}}x, $$

where *R*_f_ and $R_{\gamma i}^{{^{\text{o}} }}$ represents the induced X-ray fluorescence radiation intensity and initial intensities of the excitation photons from the Co-57 source at 122.1 and 136.5 keV, respectively; *τ*_*i*_ is the photoelectric absorption coefficient for a given photon energy and metal additive, in cm^2^/g; *μ*_i_ and *μ*_f_ are the mass attenuation coefficients for excitation photons and the secondary fluorescence radiation, respectively, in cm^2^/g; *ω* and *B* represent the K fluorescence yield and branching ratio for the transition of a specific X-ray emission photon energy; *c*_Me_ is the concentration of the metal additive in the bulk material; and d*x* represents the shield surface density increment in g/cm^2^.

Solving Eq. 1 leads to a well known two exponential formula (Eq. 2), where in case of Co-57 source, two components should be taken into consideration (due to emission of two groups of photons: 122.1 and 136.5 keV).

2 $$ R_{\text{f}} =\,   \frac{{R_{\gamma 1}^{o} \tau_{1} \omega Bc_{\text{Me}} }}{{\mu_{\text{f}} - \mu_{1} }}\left( {e^{{ - \mu_{1} x}} - e^{{ - \mu_{\text{f}} x}} } \right) + \frac{{R_{\gamma 2}^{o} \tau_{2} \omega Bc_{\text{Me}} }}{{\mu_{\text{f}} - \mu_{2} }}\left( {e^{{ - \mu_{2} x}} - e^{{ - \mu_{\text{f}} x}} } \right) $$

Equation 2 with good approximation describes behavior of the X-ray fluorescence radiation induced within the shield, that is its intensity versus thickness of the shield. For a given experimental conditions (constant geometry, photon flux, detector efficiency) and for particular shield (constant composition and exactly defined *μ*_1_, *μ*_2_ and *μ*_f_) one can observe a saturation curve of emission intensity versus thickness or curve with maximum which position is dependent on the *μ*_1_, *μ*_2_ and *μ*_f_ coefficients.

**Fig. 1:**
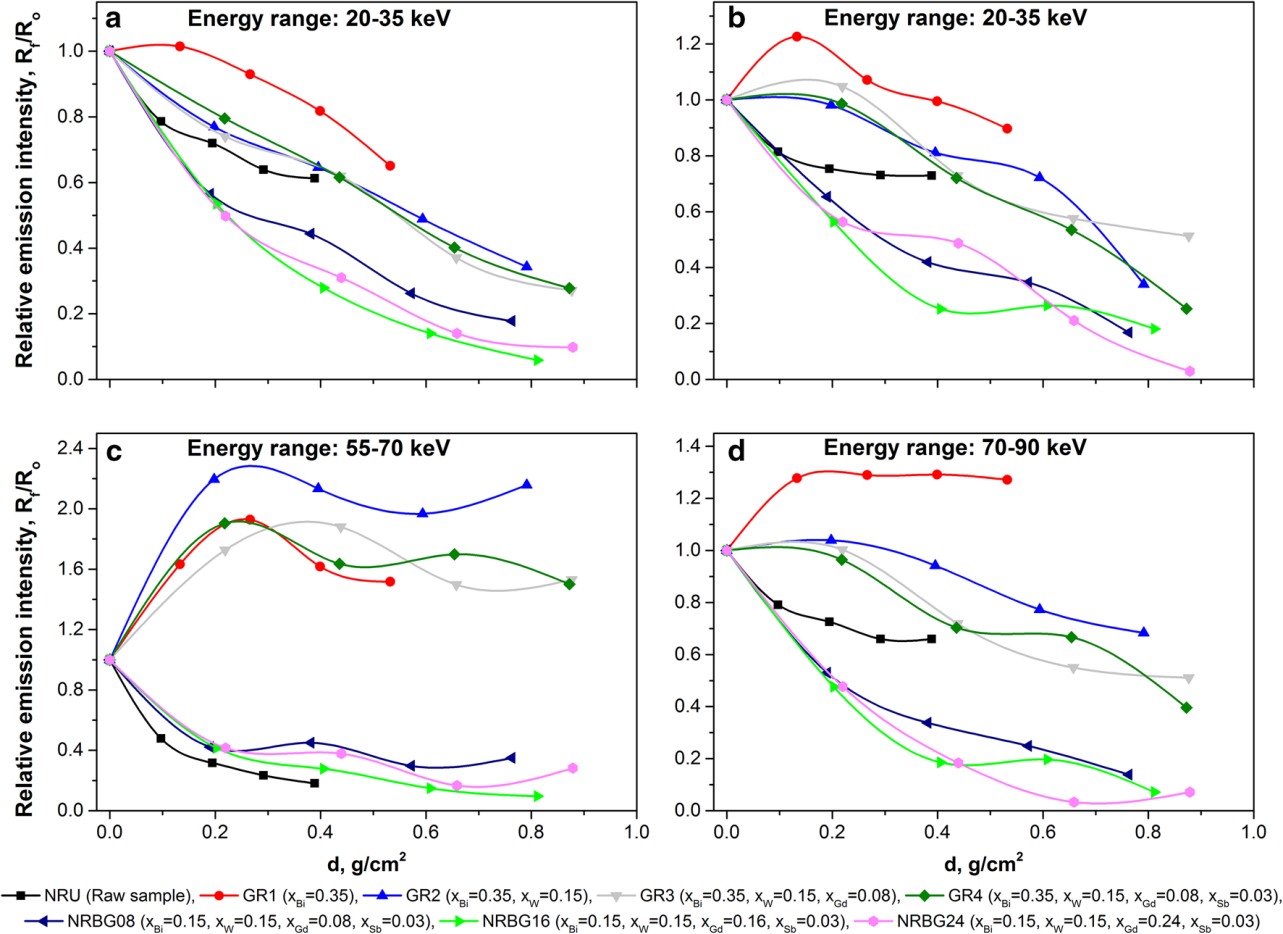
Relative X-ray fluorescence radiation intensities induced in elastomeric composites in a specific energy ranges for: **a** Sb emission (20–35 keV), **b** Gd emission (35–55 keV), **c** W emission (55–70 keV) and **d** Bi emission (70–90 keV)

Figure 1a–d present X-ray fluorescence emission intensities of the investigated shields within the specific photon emission energy ranges for Sb (20–35 keV), Gd (35–50 keV), W (55–70 keV) and Bi (70–90 keV) versus mass thickness of the particular composite. The measured photon emission intensities include scattered radiation and are background corrected in each particular energy range. Although background correction, external shield around the measurement system and application of beam collimator it was not possible to avoid completely the weak signals in each energy region originating probably from the external shield and collimator materials. What can be clearly seen, behavior of the two series of investigated composites is significantly different. In all measured energy ranges X-ray fluorescence radiation emission is much stronger for samples containing higher fraction of Bi additive (Fig. 1a–d, samples GR1–GR4, *x*_Bi_ = 0.35). It is expected as the Bi additive is the main component of the GR shields and is responsible for generation of the X-ray fluorescence radiation within the shield. Effect of the addition of lower Z elements (W, Gd and Sb) is clearly visible in GR series samples, where intensity of the X-ray fluorescence radiation gradually decreases when W, W + Gd and W + Gd + Sb additives are incorporated into the elastomeric matrix (Fig. 1b–d). Moreover, the shifts of the emission maxima towards lower thickness can be observed when moving to composite with higher heavy metal fraction, from GR1 (Bi only) to GR4 (Bi + W + Gd + Sb). One can expect, that increasing Bi concentration would result in higher attenuation effect within the shield, but from the other hand it can be also expected, that shields with higher heavy metal content (especially Bi) will generate X-ray fluorescence radiation in higher extent, what is observed as higher emission intensity within the 70–90 keV region. This is clearly visible when we take into account dependence of the X-ray fluorescence radiation emission intensity versus shield thickness for the second series of the investigated composites (NRBG series). NRBG samples consist of decreased concentration of Bi fraction (*x*_Bi_ = 0.15) as compared with the GR samples (*x*_Bi_ = 0.35), whereas W and Sb fraction were constant, *x*_W_ = 0.08 and *x*_Sb_ = 0.03 respectively. The only one variable was Gd fraction (*x*_Gd_ = 0.08, 0.16 and 0.24 for NRBG08, NRBG16 and NRBG24 respectively). The effect of decreasing Bi concentration is visible as an effective reduction of X-ray fluorescence radiation intensity in all analyzed energy regions (Fig. 1a–d). Increasing concentration of Gd additive from *x*_Gd_ = 0.08–0.24 results in further slight reduction of Bi emission intensities within the 70–90 keV energy range (Fig. 1d). Emission intensities due to excitation of W (55–70 keV, Fig. 1c), Gd (35–55 keV, Fig. 1b) and Sb (20–35 keV, Fig. 1a) and scattered radiation exhibit only small variation with Gd concentration. Generally, it can be explained, that lower emission intensities within the energy ranges of 20–35 and 35–55 keV is the result of lower fraction of scattered X-ray fluorescence radiation of Bi and W origin.

The final evaluation of the investigated shields was based on the calculation of the Dose Reduction Factor (DRF), defined as the ratio of dose deposited in the examined tissue with and without application of the protective shield (Eq. 3).

3 $$ {\text{DRF}} = \frac{D}{{D_{\text{o}} }} = \frac{{\sum\limits_{i} {R_{i} \mu_{\text{TS}} E_{i} } }}{{\sum\limits_{i} {R_{i}^{\text{o}} \mu_{\text{TS}} E_{i} } }}, $$

where *R*_*i*_ denotes radiation flux (in 1/cm^2^s), *μ*_TS_ the mass attenuation coefficient for the tissue being irradiated (in cm^2^/g) and *E*_*i*_ the energy of the absorbed photons (in keV).

DRF can be a number from the range of 0–1, and the lower DRF value corresponds to more efficient protective effect of the shield. For calculation of DRF simplified model was applied, in which dose delivered to the tissue by photons from the specific energy range was calculated assuming average photon energy *E*_*i*_ and average tissue mass attenuation coefficient *μ*_TS_. Calculated DRF coefficients for the examined composites are summarized in Table 3. The most efficient protective effect was achieved for composites with decreased concentration of Bi additive from *x*_Bi_ = 0.35–0.15, with only slight influence of the Gd concentration. The lowest DRF value (0.47), with 53 % dose reduction capability was observed for NRBG24 composite (thickness of the sample ca. 2 mm and 0.44 g/cm^2^). From the practical reasons, 2 mm shield thickness is still acceptable.

**Table 3:** Dose reduction factors (DRF) for examined composites

Composite	Additive(s)	*d* (g/cm^2^) (1 layer)	DRF^a^	DRF^b^	*d* (g/cm^2^) (2 layers)	DRF^a^	DRF^b^
NRU	–	0.097	0.92	0.92	0.194	0.91	0.91
GR1	Bi (0.35)	0.133	0.91	0.91	0.266	0.79	0.79
GR2	Bi + W (0.35 + 0.15)	0.198	0.76	0.76	0.396	0.58	0.58
GR3	Bi + W + Gd (0.35 + 0.15 + 0.08)	0.219	0.71	0.71	0.438	0.49	0.50
GR4	Bi + W + Gd + Sb (0.35 + 0.15 + 0.08 + 0.03)	0.218	0.73	0.73	0.436	0.51	0.51
NRBG08	Bi + W + Gd + Sb (0.15 + 0.15 + 0.08 + 0.03)	0.191	0.76	0.76	0.382	0.61	0.61
NRBG16	Bi + W + Gd + Sb (0.15 + 0.15 + 0.16 + 0.03)	0.203	0.72	0.72	0.406	0.54	0.54
NRBG24	Bi + W + Gd + Sb (0.15 + 0.15 + 0.24 + 0.03)	0.220	0.65	0.65	0.440	0.47	0.46

^a^X-ray fluorescence radiation of Bi, W, Gd and Sb involved

^b^Only for 122.1 and 136.5 keV Co-57 photons

Taking into account that total intensity of X-ray photons escaping from the shield is the sum of the excited fluorescence radiation (*R*_f_) and primary photons not absorbed in the shield (*R*_a_) one can write that *R*_*i*_ = *R*_a_ + *R*_f*i*_. The primary radiation flux, *R*_a_, passing the shield of thickness *x* can be described by the well known exponential dependence, *R*_a_ = *R* ^o^exp(−*μx*). From the described previously Eq. 2 and assuming for further simplification that only first part of that equation is important, one can write that intensity of the generated X-ray fluorescence radiation, within the whole energy range, can be roughly expressed by the Eq. 4.

4 $$ R_{{{\text{f}}i}} = \frac{{R^{\text{o}} \tau \omega Bc_{\text{Me}} }}{{\mu_{{{\text{f}}i}} - \mu }}\left( {e^{ - \mu x} - e^{{ - \mu_{{{\text{f}}i}} x}} } \right). $$

Then we can consider DRF coefficient also as shielding properties parameter in terms of both primary, penetrating radiation and that generated X-ray fluorescence radiation. According to Eq. 3 and introduced above simplifications, after recalculation we can write, that DRF is a function presented by Eq. 5.

5 $$ {\text{DRF}} = e^{ - \mu x} + \mathop \sum \limits_{i} \frac{{\tau_{i} \omega_{i} B_{i} c_{{{\text{Me}}i}} }}{{\mu_{{{\text{f}}i}} - \mu }}\left( {e^{ - \mu x} - e^{{ - \mu_{fi} x}} } \right)\frac{{E_{i} }}{{E^{\text{o}} }}. $$

**Fig. 2:**
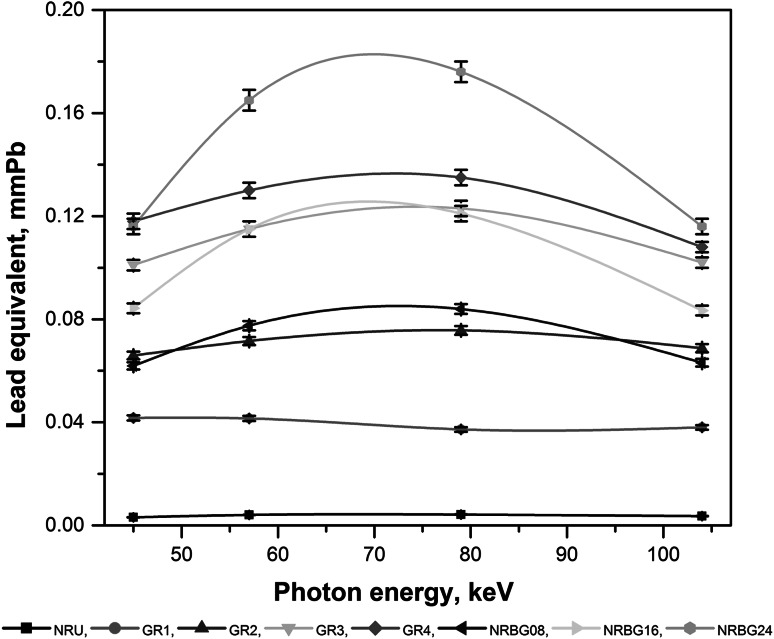
Dependence of the measured lead equivalents (in mmPb) versus X-ray photons energy for investigated elastomeric shields; relative error of each measurement ±2 %

Validity of such approach for DRF calculation has been checked for comparison of the proper lead equivalent thicknesses for investigated shields. Calculations confirmed that the highest contribution to the dose absorbed is due to the primary radiation with only slight contribution of X-ray fluorescence generated within the shield (below 10 % of the total photon flux escaping the shield). The results of measured and calculated lead equivalents values are presented in Table 4. Another presentation of these results is shown in the Fig. 2. In most cases experimentally determined lead equivalents well correspond to values obtained by calculations with higher deviations observed for GR2 sample and 79 keV photons. Measured lead equivalents thicknesses are in good correlation with calculated DRF values, where the same tendency is observed in protective properties sequence of the investigated elastomers, from NRBG24 to NRU (compare Tables 3, 4; Fig. 2).

**Table 4:** Measured and calculated lead equivalents for investigated elastomeric shields

Composite	Lead equivalents (in mmPb) for photons energy (keV)							
	45^a^	57^a^	79^a^	104^a^	45^b^	57^b^	79^b^	104^b^
NRU	0.0032	0.0041	0.0042	0.0036	0.0029	0.0040	0.0071	0.0030
GR1	0.0420	0.0420	0.0370	0.0380	0.0450	0.0460	0.0490	0.0450
GR2	0.0660	0.0720	0.0760	0.0690	0.0850	0.0870	0.1500	0.0870
GR3	0.1000	0.1200	0.1200	0.1000	0.1000	0.1300	0.2100	0.1000
GR4	0.1200	0.1300	0.1400	0.1100	0.1100	0.1400	0.2100	0.1100
NRBG08	0.0620	0.0780	0.0840	0.0630	0.0620	0.0880	0.1500	0.0590
NRBG16	0.0840	0.1200	0.1200	0.0830	0.0720	0.1300	0.1900	0.0700
NRBG24	0.1200	0.1700	0.1800	0.1200	0.0850	0.1700	0.2500	0.0840

^a^Experimental values

^b^Values calculated

**Fig. 3:**
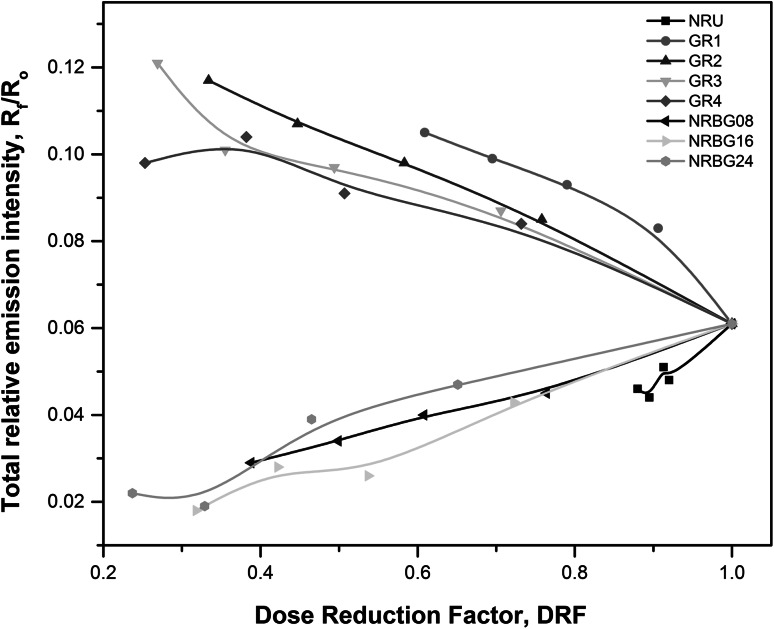
Correlation between DRF and relative total X-ray fluorescence intensity for high Bi fraction samples (GR series, *x*_Bi_ = 0.35) and low Bi content (NRBG series, *x*_Bi_ = 0.15); NRU (reference) sample without heavy metal additives for comparison

Another very interesting observation was made when comparing DRF values with the ratio of the total X-ray fluorescence radiation to total intensity. This dependence is presented in the Fig. 3. Two completely distinct behaviors of the GR and NRBG series are observed. In case of GR series one can observe opposite effect of *R*_f_/*R*_o_ and DRF: decreasing DRF (desired effect) is assisted by simultaneous increasing *R*_f_/*R*_o_ ratio (higher fraction of X-ray fluorescence radiation, undesired effect), whereas in case of NRBG series composites (NRBG08, NRBG16 and NRBG24) decreasing DRF is assisted by simultaneous decreasing of *R*_f_/*R*_o_ ratio resulting in the lower fraction of induced X-ray fluorescence radiation. The same effect as for NRBG series samples is observed also for the raw NRU sample.

The dependence of photon emission intensity within the 90–140 keV energy range vs. shield thickness is in good agreement with the exponential absorption law (data not shown) and the measured average mass attenuation coefficients are close to these calculated using XCom software and based on the elemental composition of the investigated samples. The relative error of the measured and calculated attenuation coefficients did not exceed 20 %.

## Conclusions

Eight new composites (seven containing heavy metal additives and reference elastomer matrix sample) for CT examination procedures were investigated towards their usability for shielding purposes. The best shielding properties were achieved for elastomers containing lower concentration of Bi (*x*_Bi_ = 0.15 vs. 0.35) and variable fraction of Gd additive (*x*_Gd_ = 0.08–0.24). Shielding performance of the examined elastomeric composites were determined for both characteristic X-ray fluorescence emission lines and scattered radiation. Such approach lead to a more reliable results in comparison with traditional method when only energetically narrow beam of photons is considered for a total dose delivered to the tissue. The most important fraction of the induced X-ray fluorescence photons was that originating from Bi and W additive. X-ray fluorescence radiation induced in Gd and Sb additives is of lower importance, as it constitutes of only small fraction of the total X-ray fluorescence radiation generated within the shield. Due to this fact, decreasing of Bi concentration (from *x*_Bi_ = 0.35–0.15) in the shields results in significant lowering of the total X-ray fluorescence radiation intensity both in the 55–70 (W) and 70–90 keV (Bi) energy regions as well as in the lower energy regions of Gd and Sb excitations. Calculations of the DRF values led us to conclusion that the best shielding properties exhibit NRBG24 composite. Similar results were obtained in experimental lead equivalents measurements. Next to these observations, both methods of verification of shielding properties of the investigated composites indicate for the analogical order of the protective shields performance.
